# Research on psychotherapy for refugees in Germany: A systematic review on its transdisciplinary and transregional opening

**DOI:** 10.1177/13634615231187255

**Published:** 2024-01-17

**Authors:** Annika Kleinschmitt

**Affiliations:** KomRex, Friedrich Schiller University Jena, Jena, Germany

**Keywords:** decolonizing science, migration, psychotherapy, transdisciplinary, transregional

## Abstract

Recently, an increasing amount of research has focused on adapting psychotherapy concepts for refugees moving to Germany. For a long time, research from disciplines like anthropology and cultural studies has problematized the eurocentrism of psychology's theoretical premises and methodologies. Currently, scholarship around Global Mental Health and decolonization emphasizes how knowledge production from various disciplines and regions relates to this topic and could contribute to improving respective approaches. Consequently, this review aims at evaluating the actual transdisciplinary and transregional opening of studies on psychotherapeutic interventions for refugees in Germany. It provides a theoretically as well as empirically informed basis for looking at disciplinary premises, practices, and boundaries as well as the regional locatedness of respective research. Fourteen relevant studies, published between January 1, 2007 and March 4, 2022, were identified by systematically searching the databases PubPsych and Web of Science. The studies were reviewed regarding study design, choice and characterization of target groups, regional origin and target group specific adaptations of the therapeutic concepts, integration of elements from and connections to other disciplines, and use of references to scholarship from the Global South. The findings show a pronounced focus on the development of trauma therapy approaches and moreover a broad variety of concept adaptations in response to the assumed characteristics, situations, and needs of the target groups. While the findings reveal a complex transregional pattern of development and adaptation of the therapeutic concepts, transdisciplinary opening and reference to the Global South appear scarce.

## Introduction

During the last decades, a steadily increasing amount of research has focused on developing and adapting mental health care approaches, and especially psychotherapeutic concepts for refugees moving to Germany (Borbé et al., 2009; Bozorgmehr et al., 2016; Hoell et al., 2021; Machleidt & Sieberer, 2013). Respective treatment concepts get subsumed under various umbrella terms, depending on the context and setting of treatment, the disciplinary backgrounds of the researchers, historical influences, geographical locatedness as well as the underlying definitions of culture, belonging, and identity. If approached from a clinical psychology perspective, these range from “transcultural psychotherapy” and “intercultural psychotherapy” to “cross-cultural counseling” and “culture-sensitive psychotherapy.” When located within Anthropology, Cultural Studies, or Sociology, subdisciplines are named Psychological Anthropology, Postcolonial Health, Sociology of Mental Health, or Sociology of Health and Illness.

For a long time, the latter fields have criticized that the psy-disciplines rely on epistemologies and methodologies which are deeply rooted in European traditions and biased by colonial legacies (Antić, 2022; Bowker & Star, 2000; Bracken et al., 2021; Kleinman, 1977; Parker, 1999) and thus far from universal. At the core of this critique, it has been argued that in clinical psychology “culture” is not considered an inherent part of the psy-disciplines in general, but instead is used to mark and define the discipline's non-Western Other (Derrida & Nicholson-Smith, 1991; Long, 2017; Pillay, 2017; Makhubela, 2016). In applied research on psychotherapeutic interventions, terminology, concepts, and knowledge only partially relate to current developments in cultural theory. Instead, they are often either inherited from the body of literature of Western psychology and psychiatry, or are spontaneously recombined and used interchangeably (Steinhäuser et al., 2014). Furthermore, research and concepts from the Global South remain mostly absent from reference lists—at most, they are taken into account on the theoretical basis of a cultural comparison framework which essentializes culture and particularizes any knowledge and even research from respective regions. As a result, a narrow definition of “culture” is established, which primarily associates it with ethnic categories and national origin (Antić, 2021; Kirmayer, 2006) and thereby enfolds a discursive power, which manifests essentializations and obliterates discourses on other aspects of identity and structural inequalities like socioeconomic disparities, political rights, and racism (Bhabha, 2012; Comaroff, 1978; Hountondji, 2002; Spivak, 1990).

In some cases, and usually fragmentarily, these discourses have started to enter parts of clinical scholarship (Calliess et al., 2012; Wohlfart et al., 2006) and more transdisciplinary approaches have started to emerge (Heyken et al., 2019; Nguyen et al., 2021). By turning from intercultural to transcultural psychotherapy, awareness has risen that “culturally competent” health care itself can become discriminating and stigmatizing if underlying conceptualizations of culture remain narrow and static, and that consequently more participatory therapeutic concepts and a focus on mutual understanding and reflection of positionality are necessary (Kleinman & Benson, 2006; Kluge, 2014; Özbek & Wohlfart, 2006).

Especially since around 2015, when the numbers of refugees migrating to Germany increased as a consequence of the war in Syria and the strengthening of the Taliban in Afghanistan and IS in Iraq, these debates have gained considerable influence beyond cultural studies, including debates about psychotherapy for migrants. Refugees became the main target group of psychotherapeutic interventions, and while cultural sensitivity faded into the background, processes of exceptionalization and Othering gradually shifted and seemed to define a new target group, which is primarily characterized as displaced, war-affected, and potentially traumatized (Fassin & Rechtmann, 2005). From a pragmatic clinical perspective, the immediate necessity to provide psychotherapy for war-affected refugees increasingly takes priority over sophisticated elaborations on the exact definitions of concepts and categorization into subdisciplines. Though the need for applied therapeutic concepts is not in question, this new focus sustained—or possibly even deepened—the construction of refugees as Other patients, while at the same time leaving psychology's theoretical concepts and methodological approaches intact.

In summary, disciplinary premises and practices are inseparably tied to a European history. They have the power to significantly shape the explanatory basis on which symptoms are discerned and subsumed into syndromes and disorders. They influence what is defined as “cultural” and what is conceived as contextual, social, or socioeconomic; which kind of knowledge is considered scientific and enters the body of research; and which treatment options are evaluated as suitable for whom, delivered by whom, and under which circumstances. Concurrently, the locatedness of concepts within specific regional and socio-spatial contexts remains invisible and often implicitly eurocentric. Although these issues have been increasingly debated and problematized, there is still ample need for research which (1) sensibly and carefully connects existing branches of knowledge production and (2) provides empirically grounded approaches to evaluation and implementation. In line with these needs, the theoretical part of this article addresses the first issue by connecting current attempts of decolonizing GMH (Global Mental Health) to postcolonial theory. The subsequent literature review presents one way in which this theoretical critique can be actively used to evaluate and improve the empirical basis of research on psychotherapy.

## Transdisciplinarity and transregionality as current paradigmatic shifts? Between global mental health and decolonization

With the emergence and current spread of GMH, these questions found a new entry point to gain relevance for policymaking and research. When in 2001 the WHO first evaluated the provision of comprehensive access to mental health care on a global level as a high priority (WHO, 2001), a paradigmatic shift for mental health research was initiated. In the following years, an increasing number of projects in low- and middle-income countries were launched, to compensate for the comparably lower number of clinical trials and intervention studies (Lund, 2020) and eventually to improve health equity and the provision of comprehensive access to mental health care on a global level (Burgess et al., 2020; Collins, 2020; Lund et al., 2011; Patel et al., 2007). Especially since 2015, the promotion of mental health became part of the Sustainable Development Agenda, and the close connection between the implementation of prevention and treatment programs and the development goal of “closing the treatment gap” between high- and low-income countries became increasingly visible (Patel et al., 2018; WHO, 2014, 2020). Consequently, GMH was and is harshly criticized for its neoliberal and neocolonial development agenda, its depoliticization of illness and care structures, and its masked-as-universal eurocentrism (Das & Rao, 2012; Mills & Fernando, 2014; Summerfield, 2012). While on the one side, GMH reinforces neocolonial structures of knowledge production, on the other it thereby constitutes a new entry point for decolonizing the discipline by shedding light on the powerful and manifold effects of globalization on the psy-disciplines.

From the very other end of the disciplinary spectrum, the literary theorist Gayatri Chakravorty Spivak has described this predicament from a feminist and postcolonial perspective. Using the examples of Comparative Literature and Cultural and Postcolonial Studies, she describes how globalization and migration movements challenge disciplinary boundaries and influence disciplinary shifts or even the founding of new disciplines (Spivak, 2003, p. 3). She emphasizes that although, in view of human history, migration is nothing new or exceptional, at the current moment individual experiences—and especially those of migrants—become increasingly less detachable from globalization and imperial leverages (Spivak, 2003, p. 85). In the struggle to deal with these effects of globalization, she states:

We cannot and should not reject this impulse toward generalization, which has something like a relationship with globalization. If we do – and some have the ignorance and luxury to do so – we will throw away every good of every international initiative. The other side – the side of capital – will not (and cannot) throw away the power of the move toward the general. (Spivak, 2003, p. 46)

Following Spivak's perspective, GMH would be more than a dominant narrative, which needs to be countered along the line of its own logic by detaching psychotherapy concepts from globalization. Rather it constitutes a paradigmatic shift, which reacts to globalization (and, e.g., its impact on migration), thereby raising the psy-disciplines to a global level and opening a discursive space for negotiating new directions for these disciplines. This space already started to open transregionally and transdisciplinary and the crucial matter is to decide which disciplines and regions will be included, precisely to prevent GMH from further shifting into the direction of apparently universal eurocentrism and thereby further entrenching a manifestation of unequal power dynamics. Whereas reasons and motivations to promote health care on a global level as well as approaches to implementation differ tremendously among various actors, the necessity of taking globalization into account has increasingly become a common issue (Lovell et al., 2019; Peseschkian, 2017).

Some of the debates which arose around GMH during the last years have already started to fill this space by connecting it to current attempts of decolonization. For example, this trend is reflected by scholarship which stresses that addressing these challenges necessarily requires increasing inter- and transdisciplinarity, diversifying sources of knowledge, and recognizing and changing (neo)colonial power dynamics and persistent socioeconomic inequalities (Chibanda, 2017; Cox & Webb, 2015; Kirmayer & Pedersen, 2014). In order to achieve GMH's pragmatic goals of “closing the treatment gap,” the complex challenge of actually achieving epistemic justice and plurality must not be underestimated. It requires close attention to patients’ experiences and allegedly “un-scientific” local approaches to Mental Health and healing (instead of overlooking them as part of the gap) as well as a shift of focus from adapting therapeutic models to focusing on the adaptation practices themselves (Bemme & Kirmayer, 2020; Di Nicola, 2020; Kirmayer, 2012; Sax, 2014).

In summary, these critical approaches to mental health underline that connecting different branches of knowledge production requires a continuous reconnection to critical alertness, and that the simultaneous fostering of divergence constitutes an indispensable prerequisite. The assumption that some of GMH's goals can be compatible with decolonization must not serve as a hall pass for appropriation of the latter. Thinking of global levels as indispensable elements of local analyses and conducting negotiations on mental health on a global level has not to be equalized with exclusively looking for all-encompassing one-size-fits-all solutions. Recognition of assimilation, acceptance of incommensurability, and the rejection of specific aspects or even entire branches of GMH, as well as the adding of new approaches by critical scholarship and activists have to remain genuine options. Critique needs to take place on different levels, which encompass local *and* global as categories for analysis and resistance, but which at the very same time move beyond the dichotomous logics of “western” and “non-western” (Spivak, 1994), “specific” and “universal” (Spivak, 1990), or “local” and “global” (Bemme & D'Souza, 2014). For example, Bajbouj and colleagues emphasize in a recently published editorial that with inequalities resulting from barriers to access to health care for migrants and insufficient facilitation of health (system) literacy, some of the issues and goals of GMH likewise constitute a crucial matter in high-income countries like Germany (Bajbouj et al., 2021; see also Hajak et al., 2021).

## Aims and scopes of this review

Taking these current developments on several levels into consideration, the present review explores possible pathways for deliberate synthesis of diverging fields of research. In analogy to the German concept of “intercultural opening,” which aims at improving migrants’ access to regular care structures (Penka et al., 2012), it assesses the openness of German psychotherapy research. For that purpose, it analyzes how researching along the lines of disciplinary definitions and concepts influences the design of study projects and thereby eventually the provision of psychotherapy for refugees in Germany. To do so, the review looks at each study's design and choice of target groups, and examines what each study characterizes as particular about their target group (e.g., cultural differences, language barriers, socioeconomic conditions, flight biographies, residence status, and legal situations). Furthermore, it explores which therapeutic concepts were applied in the study and how they were adapted in order to fit the (assumed) needs of the target groups (e.g., culture- and language-related adaptations, adaptations in response to migration biographies). On the level of transdisciplinarity, it assesses if the studies mention the use of theories, methods, and findings from other disciplines than psychology and psychiatry, and if the therapeutic concepts are designed interdisciplinarily. A complementary, transregional focus is on evaluating the use of references to research from the Global South within the body of research on psychotherapy for refugees in Germany and the regional origin of the therapeutic concepts.

The project is designed as a review in order to build upon a comprehensive empirical basis when looking at transdisciplinary intersections and frictions within respective research. Its further future objective is to provide an informed groundwork for analyzing the challenges, problems, and opportunities which arise from disciplinary rigor on the one hand as well as transdisciplinary opening on the other.

## Methods

### Inclusion and exclusion criteria

Included were randomized controlled trials as well as pilot studies without a control group, which comprised a psychotherapeutic intervention as a main component and gave a replicable and transparent description of the psychotherapeutic procedure. Included studies took at least two measurements of psychological distress, one before and one after the intervention. The target groups of the studies specifically included persons who 1) suffered from psychological distress at the beginning of the intervention and 2) had migrated to Germany at any point in their lives and lived there during the intervention period. Germany was chosen as a starting point since it represents one among other (European) immigration countries. In view of the fact that each of these countries differs with regard to immigration and health care policies and builds on different systems of health-related classifications and systematizations, which also affects the way in which research projects are developed and implemented (Scott et al., 2014), inclusion was restricted to one country. Since Germany is one of the countries in which respective research on the mental health of refugees has increased significantly over recent years, there is a need for an in-depth analysis of respective research's premises and practices.

Excluded were multinational and multicenter studies, as well as those which did not mainly focus on psychotherapeutic interventions but instead on any other mental health-oriented intervention (e.g., prevention programs, social work).

### Search strategy

The databases PubPsych and Web of Science were systematically searched for relevant publications on March 4, 2022. The focus was on current research, so the publication date was limited from January 1, 2007 to March 4, 2022. It should be noted that few intervention studies on psychotherapy for refugees in Germany had been conducted before these dates (Bozorgmehr et al., 2016). The following search details were used in both databases:

((migrant OR migrant* OR migrants OR migrational OR migration OR refugees OR refugee OR refugee* OR cross-cultural OR cross-cultur* OR culture-sensitive OR culture-sensitiv* OR transcultural OR transcultur* OR intercultural OR intercultur*) AND (psychotherapy OR psychotherapy* OR psychotherapeutic OR psychotherapeut* OR counseling OR counseling* OR mental health care OR mental health care*) AND (germany OR german OR german*))

**Figure 1.**
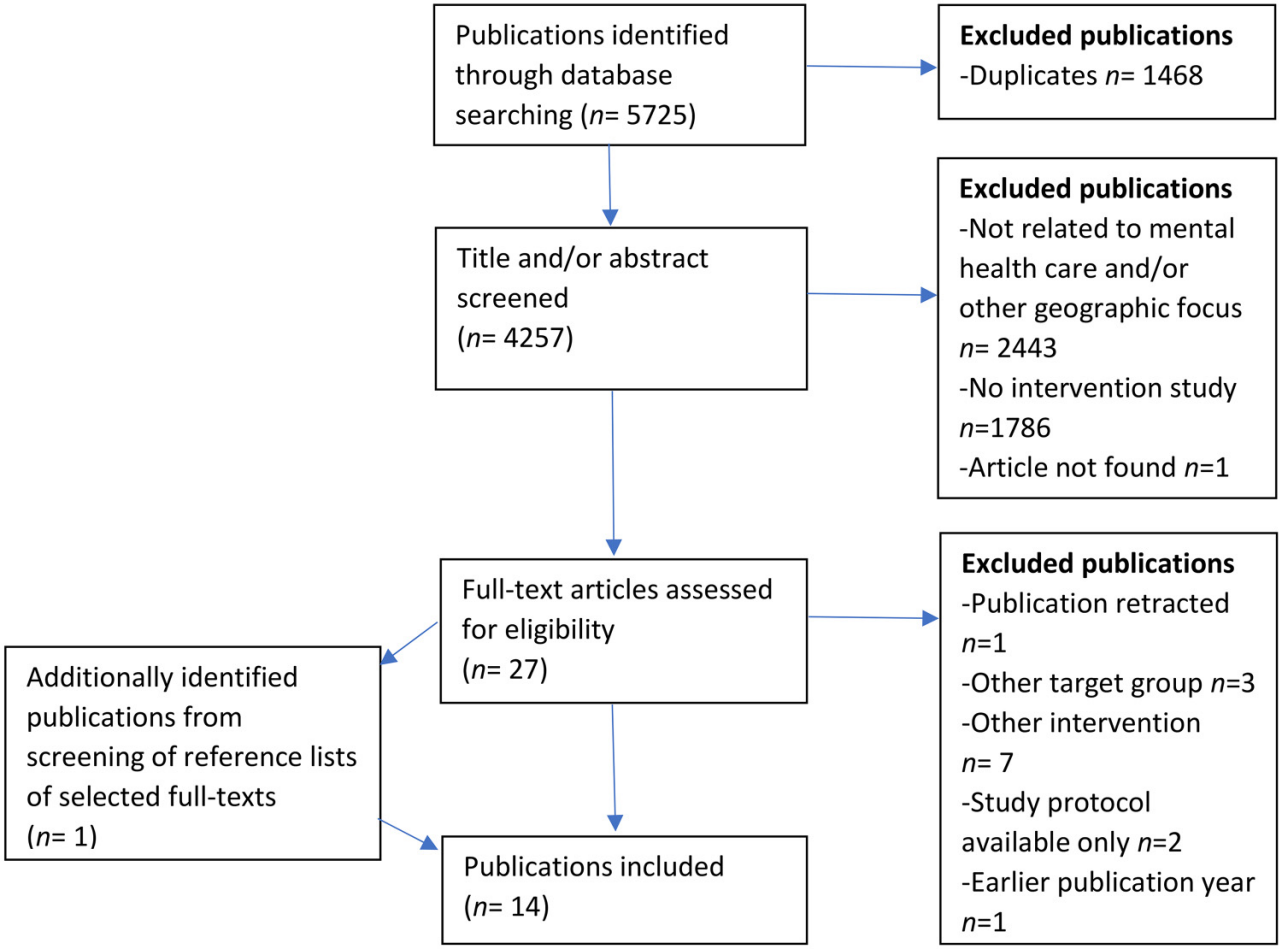
Flowchart of the screening and selection process.

Publications in English and German were considered, and accordingly the search was repeated with a German translation of the search details. In the next step, duplicates were removed, and all remaining publications were screened for relevancy following a two-step screening process (see Figure 1). In accordance with the inclusion and exclusion criteria, the first step involved screening of the title and abstract. If the geographic location of the study population and authors was not evident from the abstract, the information was extracted from the full text and/or from additional research on the study group. In the second step, full texts were analyzed, including the screening of their reference lists for additional relevant publications. Due to the current lack of similar research in the field, the analytical strategy was primarily explorative and qualitative (except for “References to scholarship from the Global South”) and aimed at exploring a broad range of different domains. Domains of analysis were pre-defined according to the preceding theoretical considerations and as described in the next two sections. Subsequently, the selected studies were read in detail and all content which fitted with the pre-defined domains is listed in the tables in this article.

### Assessment of transdisciplinary opening

Rigorously restricting the methodological and regional scope in a review that assesses transdisciplinary and transregional opening might seem paradoxical. While any definition of inclusion and exclusion criteria contributes to the artificial consolidation of “the discipline of clinical psychology,” it nevertheless is inevitable to define a starting point in order to analyze and understand the discipline's practices and boundaries. Studies on psychotherapeutic intervention were chosen as objects of investigation since they represent a core part of applied clinical research and furthermore give detailed information on the methodological procedure as well as a clearly defined description of the therapeutic concept. The focus is therefore not on explicitly transdisciplinary research, but on the transdisciplinary opening of applied clinical research.

As a precondition for evaluating transdisciplinary opening, disciplinary practices of psychotherapeutic intervention studies were analyzed. This included the choice and restriction of target groups (inclusion criteria), the characterization of target groups by the authors of the study, and the target group-specific adaptations of the therapeutic concepts.

Transdisciplinary opening was assessed along three categories. The first category contains the use of theory from other disciplines, especially cultural and anthropological theory. The second contains the transdisciplinary opening of the study procedure and methodological approaches, i.e., the use of methodology which goes beyond psychometric effect-measures of symptom improvement. The third contains elements of the therapeutic concepts which transgress the boundaries of psychotherapy, i.e., the inclusion of any elements which are not part of psychotherapeutic interventions in a strict sense but draw on other disciplines (e.g., social work, medicine), the inclusion of professionals from other disciplines, and the engagement of psychotherapists beyond psychotherapy.

### Assessment of transregional opening

Similar to transdisciplinarity, transregionality was analyzed on several levels. On the level of study design, references to scholarship from the Global South were counted, while on the level of therapeutic content, the transregional development of the therapeutic concepts was traced.

Reference to scholarship from the Global South was examined in order to render visible power imbalances in academic knowledge production and epistemic injustice (Collyer, 2016; Di Nicola, 2020). Self-evidently, the “the Global South” is no homogenous category. Rather, it merges geographic locatedness, geopolitical power relations, economic inequalities, colonial history, epistemic injustice, and questions of subalternity and cultural differences into two seemingly stable and dichotomous categories (Dados & Connell, 2012; Kloß, 2017). As a result, subsuming many diverse regions into one unified category follows a structure of counter-narrativity, which means that it remains bound to categories and narratives which have historically evolved in the Global North (Spivak, 1994). This is necessary in order to render them visible at all vis-à-vis the Global North, but at the same time runs the risk of further marginalizing the most marginalized narratives within this general category. As a consequence, concretely delineating the Global South consequently represents a “strategic” (Spivak, 1990), more than difficult, epistemically violent, and always preliminary attempt to respond to global inequality. Since this study aims at shedding light on already long-lasting structural inequalities and epistemic injustice (see also Confraria et al., 2017; Haug, 2021), the Global South was defined based on the list of countries of the Group of 77 and China (Group of 77 at the United Nations, 2022). Scholarship was counted as “from the Global South” when at least one author of a publication was affiliated with an institution in the Global South.

It is clear that this country-based categorization remains a preliminary and insufficient criterion, especially since it must be assumed that some persons in institutional structures in countries which are classified as Global North work and research according to conditions which resemble power structures of the Global South, as well as the other way around. Furthermore, not all authors who have migration backgrounds themselves or have been born, raised, or educated in the Global South are still affiliated with institutions there. An additional focus on analyzing reference to scholarship from countries of origin of the respective target groups was discarded, since, first, not all studies assessed “country of origin” as a relevant category and second, it is likely that a considerable proportion of participants identify with minorities which might not necessarily be well represented by research from respective “countries of origin.” And finally, it must be noted a lot of knowledge production on mental health happens out of sight of institutions and e.g., not all persons, who contribute to a study project might appear in the list of authors.

On the level of therapeutic content, the regional origins of the therapeutic concepts which were used in the study projects were identified. Furthermore, it was analyzed in which countries concepts were adapted and knowledge from which regions substantially influenced the concepts. Similar to the analysis of reference to scholarship from the Global South, information was structured by using nation-based categorizations.

## Results

Initially, 5,725 records were identified via database search. After the removal of duplicates and the application of inclusion and exclusion criteria, 27 publications were assessed as possibly eligible. Full-text and reference list screening of selected full texts led to the identification of one additional relevant publication, which had not been identified via database search. Fourteen publications were excluded during full-text screening (for detailed information, see Figure 1). Finally, 14 publications were selected for analysis. In line with the aims of the review, the studies were analyzed regarding their general characteristics, the characterizations of their target groups, and their choice and target group-specific adaptation of therapeutic concepts. Furthermore, the transdisciplinarity of the studies as well as of the therapeutic concepts was analyzed. Similarly, transregionality was assessed, first operationalized as reference to scholarship from the Global South, and second as an analysis of the origin and transregional development of the therapeutic concepts.

Due to the different settings, target groups, inclusion criteria, and backgrounds and work experiences of the therapists, the study results (in the sense of treatment effects) can be considered not comparable and will not be further discussed. However, all studies reported symptom improvement (for detailed information, see supplementary material, Table 1).

### Characteristics of the studies

Of the included studies, six are randomized controlled intervention studies (one with two treatment conditions [2], three with waiting group [4, 6, 10], two compared to treatment as usual [7, 9]), while the other eight are pilot studies without control group. Sample sizes range from seven to 76 participants per treatment condition (M = 19.27, SD = 17.33), with drop-out rates / incomplete participation rates between 0 and 57% (M = 23.83, SD = 18.79).

### Target groups

Regarding the target group, the majority of studies focused on adults. Five study projects explicitly mentioned adult age as inclusion criterion (1, 3, 4, 7, 12, 14), while two studies defined youth and/or young adults (6, 13) and two studies children (8, 10) as their target group. Two did not mention age as an inclusion criterion, but seemed to focus on adults (2, 5).

All studies specified refugees and/or asylum seekers as their target groups. Three studies included only participants from specific countries, namely Afghanistan (6), Bosnia (7), and Turkey (5). Two studies from the same working group included only participants who spoke specific languages, namely Dari or Farsi (3, 4). The other nine studies included participants independent of geographical origins.

In their introductory parts and target group descriptions, all studies pointed out that refugees had potentially experienced traumatic events and/or that the population showed a high prevalence of PTSD and other disorders, which have been caused by pre-, peri-, and postmigratory stress. Particularly listed as postmigratory stressors were structural and legal barriers (1, 6, 9, 11, 13), integration-related stress (1, 9, 10, 13), and insecure residence status (2, 3, 4, 6, 7, 8, 9, 11, 12, 13, 14). Half of the studies mentioned culture- and language-related barriers to accessing care, and/or ascribed to their target groups culturally different concepts of distress and culturally specific symptoms (3, 4, 5, 6, 7, 9, 13).

In line with the choice and characterization of the target groups, most studies focused on treating PTSD and trauma-related symptomatology. In eight studies (2, 5, 7, 9, 10, 12, 13, 14), a PTSD diagnosis was a necessary inclusion criterion. One study included only patients who showed symptomatology of a posttraumatic stress response (8). The other five studies (1, 3, 4, 6, 11) were designed transdiagnostically and included for example affective, somatoform, anxiety, PTSD, and eating disorders. Out of these, one set the additional inclusion criteria of having experienced at least one potentially traumatic event (6) and one included only patients who had experienced torture or war-related violence (11).

### Choice and adaptation of the therapeutic concepts

All studies chose their therapeutic concepts based on the assumed characteristics and situations of their participants and adapted their concepts according to their assumed needs (see supplementary material, Table 2).

#### Choice of therapeutic concept and focus

Two studies (5, 11) were embedded in multimodal inpatient settings, while the other 12 were conducted in outpatient settings. Almost all therapeutic concepts were located within CBT, while one (1) was developed based on interpersonal therapy. In accordance with the predominant focus on PTSD and trauma-related symptomatology, the majority of studies focused either on applying techniques to deal with trauma-related symptomatology or exposing patients to trauma-related memories. Two of the trauma-oriented studies especially highlighted that they applied concepts which meet the needs of patients with biographies of multiple and repeated traumatization (2, 9).

In some studies, residence status served as a criterion for reasoning the choice of the therapeutic concept. Three studies reasoned for the use of primarily stabilizing techniques (CPT, first phase of trauma-specific psychotherapy, imaginative stabilization techniques) on the basis of unstable living conditions and insecure residence status (7, 12, 14). The studies which applied exposure techniques (EMDR-based group therapy, TF-CBT, KIDNET, phase model of trauma exposure, NET) did so due to the higher effectiveness of these concepts and in spite of in some cases only temporary or possibly insecure residence status (8, 9, 10, 11, 13). One study used a narrative- and trauma-focused but not directly exposure-based technique (KNTT), based on the explanation that many cultures are not used to confrontative trauma therapy (5). Only one study directly compared stabilizing techniques (SIT) to exposure (NET) (2).

Of the studies which did not specifically focus on PTSD, three (3, 4, 6) used stabilizing emotion-regulation techniques (CA CBT, CA CBT+, STARC) in order to cover the variety of disorders which can result from experiencing trauma-related distress, and two specifically focused on improving integration and dealing with post-migration stress (IITF, CA CBT+) (1, 4).

#### Culture- and language-related adaptations

Five studies reported that they specifically adapted the therapeutic process and therapeutic material to make them more culturally specific (3, 4, 5, 6, 7). These cultural adaptations included the use of culturally relevant metaphors, visualizations, and examples (3, 4, 6, 7), working with culture-related explanations of causes and convictions about the traumatic event (3, 4, 7), a special focus on family and community (3), a non-judgmental attitude of the therapist, and encouraging to take the cultural contexts into account before judging a behavior and the integration of relevant resource persons (6). One study focused on using a culturally adapted narration structure for exposure (5). All of these were the same studies which had restricted their target groups to specific geographic origins, and accordingly, culture was primarily defined on the basis of national origin (5, 6, 7) or common language (4, 5). Of the other studies, one aimed at cultural adaptation by training therapists in general cultural sensitivity (12), and two provided culture mediation (1, 13).

Five studies conducted therapy with therapists who spoke one of the participants’ first languages (3, 4, 5, 7). Of the other studies, one conducted therapy in English (14), one offered the possibility of code-switching (8), one used simple language (6), and the other seven provided the possibility of language mediation (1, 2, 9, 10, 11, 12, 13) One provided the possibility for participants to choose the sex of the interpreter (13) and one provided female interpreters for female participants (9).

#### Adaptations in response to flight and migration

In addition to the choice of concepts, a broad range of conceptual adaptations was realized in response to different aspects of flight and migration. One study's therapeutic concept took into account suffering which was caused by individual as well as collective traumatization (5). One study explicitly included the possibility of crisis intervention in reaction to acute post-migratory stress (11). One study mentioned the use of examples which are typical for refugees’ situations (12). Three of the studies which treated children and youths were designed in such a way that the involvement of parents was not necessary, in case these were absent (13) or possibly occupied with their own psychological distress (8, 10). One study, which aimed at treating unaccompanied refugee minors, included the development of a safety plan to react in case of refusal of asylum and the possibility to add grief-specific components (13).

#### Adaptation of questionnaires and therapy material

Most studies used questionnaires which had been adapted to the respective target group in various ways. Applied questionnaires were translated (8), back-translated (6, 11), “already available” in the respective language (1), partly (back)-translated, partly validated in respective languages (3, 4, 5), or cross-culturally adapted (14). In two studies, only part of the questionnaires was adapted, by translation (7) or by adding of target group-specific items (13). Three studies explicitly mentioned the use of translated therapy material (3, 4, 12), while one mentioned the use of cross-culturally applicable material (8).

### Transdisciplinarity

#### Use of theory from other disciplines

None of the selected studies included explicit references to cultural or anthropological theory. One study historically and paradigmatically classified the therapeutic concept as rooted in the constructivist turn and systemic therapy and gave a short description of the underlying theoretical premises and parallels with other therapeutic approaches (5).

#### Transdisciplinarity of the study procedure

Not explicitly beyond the boundaries of clinical psychology, but still extending the margins of the methodological procedure of conducting an intervention study, four studies provided additional qualitative analysis and/or process reflections, either within the same paper or as an additional evaluation paper. One study (12) supplemented the effect measures with case studies of the therapy processes as well as open question-based evaluative interviews with patients (*n* = 11) and therapists (*n* = 6). The primary aim was to identify helpful intervention elements and further evaluate the reasons for the high drop-out rates in the study. Similarly, but separately published, Zehetmair and colleagues evaluated their psychotherapeutic group intervention (14) by conducting 30 qualitative interviews on symptom improvement, difficulties with the study procedure, and participation (Zehetmair et al., 2019). Following up on the two culturally adapted CBT studies by Kananian et al. (3, 4), the working group provided an additional paper with a detailed reflection on the process of cultural adaptation. In preparation, a focus group was conducted with patients who had participated in the intervention study. The paper gives a detailed description of the origin and sources of the study material, the background of the authors, the culture-specific knowledge used in the study, and a reflection on the definition of the target group and on the translation and adaptation process (Kananian et al., 2021).

#### Transdiciplinarity of the therapeutic concepts

The transdisciplinarity of the therapeutic concepts and procedures encompassed multiprofessionality, supplementing the therapeutic content with elements from other disciplines, and enhancing current safety (see supplementary material, Table 2). Beyond the already described involvement of language and cultural mediators, multiprofessionality ranged from the involvement of volunteer workers (12) or caregivers (8, 13) to multimodal inpatient treatment (5, 11) with e.g., medical, psychiatric, and social treatment services as well as body and creative therapeutic modules. One integration-focused study explicitly developed an outpatient setting, which included social work, facultative occupational therapy, and psychiatric treatment (1). Two studies included transdisciplinary elements directly in the therapeutic concept, by supplementing it with meditation and yoga-like exercises (3, 4).

Another aspect, which was evaluated as extending the boundaries of psychotherapy, was the attempt to enhance safety by improving the participants’ legal situations. With testimony therapy as a model, three studies mentioned that they handed out written biographies (2, 9) or documents (10) to the participants after study participation. In two of these studies, it was specified that participants could use these during the asylum process, e.g., by submitting these to the court and/or human rights organization to prove human rights violations (9, 10). One other study underlined temporary safety, due to the fact that participants were for the most part safe from deportation during treatment/study participation (7).

### Transregionality

#### Reference to scholarship from the Global South

The number of total references in the selected publications ranged from 33 to 71 (M = 44.71, SD = 11.82). Besides publications with explicit authorship (mostly journal articles), these contained references to publications, manuals, guidelines, and laws without clearly attributable authorship, which were published by national (M = 3.4%, SD = 2.9%) or international (M = 2.0%, SD = 2.3%) organizations and institutions. Of the 14 studies, 12 included references to scholarship of which at least one author is listed as affiliated with an institution in the Global South (see Table 1). The proportion of references to this scholarship ranged from 0 to 22.4%, with an average of 8.6% (SD = 7.0%). In total, the 14 studies listed 626 references, of which 58 (9.3%) are authored or co-authored by at least one author who is affiliated with an institution in the Global South, while 537 (85.8%) of the references are entirely authored by persons who are affiliated with institutions in the Global North. The remaining proportion distributes to references to publications, manuals, guidelines, and laws, published by national (19; 3.0%) and international (12; 1.9%) organizations and institutions. In total, 20 different countries of the Global South are represented in the reference lists of the 14 publications.

**Table 1.** References to scholarship from the Global South and from national and international organizations.

**Study**	**Total references**	**Scholarship with ≥1 author, affiliated with an institution in the Global South (total/percentage)** **^a^**	**Publications, manuals, guidelines, and laws by national organizations and institutions (total/percentage)**	**Publications, manuals, guidelines, and laws by international organizations and institutions (total/percentage)**
**Brakemeier et al. (2017)** **[1]**	33	0	4/ 12.1 Ärztekammer (1), DGPS (1), 1 Senat (1), SGB (1)	1/ 3.03 WHO (1)
**Hensel-Dittmann et al. (2011)** **[2]**	37	3/ 8.1 Bosnia-Herzegovina (1), China (1), Uganda (1)	1/ 2.7 APA (1)	1/ 2.7 VIVO (1)
**Kananian et al. (2017)** **[3]**	44	8/ 18.2 Afghanistan (1), Iran (5), Nepal (1), South Africa (1)	1/ 2.3 BAMF (1)	0
**Kananian et al. (2020)** **[4]**	67	15^a^ / 22.4 Afghanistan (1), China (1) India (2), Iran (4), Iraq (1), Kenya (2), Lebanon (1), Nepal (1), Pakistan (2), South Africa (1)	1/ 1.5 APA (1)	1/ 1.5 UN (1)
**Kizilhan (2010)** **[5]**	38	0	1/ 2.6 MFH (1)	0
**Koch et al. (2020)** **[6]**	40	2/ 5 Iraq (1), Lebanon (1)	1/ 2.5 German Federal Office (1)	0
**Kruse et al. (2009)** **[7]**	34	5/ 14.7 Bosnia-Herzegovina (2), Lebanon (1), Mozambique (1), Uganda (1)	1/ 2.9 APA (1)	0
**Lempertz et al. (2020)** **[8]**	36	1/ 2.8 Syria (1)	2/ 5.6 APA (1), BAMF (1)	3/ 8.3 ISTSS (1), WHO (2)
**Neuner et al. (2010)** **[9]**	40	5/ 12.5 Bosnia-Herzegovina (1), Chile (1), China (1), Malaysia (1), Sri Lanka (1)	2/ 5 APA (1), NICE (1)	1 / 2.5 WHO (1)
**Ruf et al. (2010)** **[10]**	43	6/ 14 Chile (1), Iran (1), Sri Lanka (1), Uganda (3)	1/ 2.3 APA (1)	0
**Stammel et al. (2017)** **[11]**	54^b^	1/ 1.9 Nepal (1)	1/ 1.9 APA (1)	1/ 1.9 WHO (1)
**Steil et al. (2021)** **[12]**	50	4/ 8 China (1), Congo (1), Iraq (2)	0	2/ 4 WMA (2)
**Unterhitzenberger et al. (2019)** **[13]**	39	1/ 2.6 Zambia (1)	2/ 5.1 NICE (1), Phoenix Australia Center (1)	1/ 2.6 APA (1)
**Zehetmair et al. (2018)** **[14]**	71^b^	7/ 9.9 Bosnia-Herzegovina (1), El Salvador (1), Iran (1), Iraq (2), South Africa (1), Uganda (1)	1/ 1.4 BAMF (1)	1/ 1.4 UN (1)

The table shows the total number of references of each publication. Further, it shows total numbers and percentages of publications with ≥1 author, affiliated with an institution in the Global South and publications, manuals, guidelines, and laws published by (a) national and (b) international organizations and institutions.

^a^ If in one publication, two authors were affiliated with institutions in two or more different countries of the Global South, these were still only counted once in the total score.

^b^ The list of references included additional references to software, which were excluded from the analysis.

Abbreviations: APA: American Psychological Association; BAMF: Bundesamt für Migration und Flüchtlinge; DGPS: Deutsche Gesellschaft für Psychologie; ISTSS: International Society for Trauma and Stress Studies; MFH: Medizinische Flüchtlingshilfe; NICE: National Institute for Health and Care Excellence; SGB: Sozialgesetzbuch; UN: United Nations; WHO: World Health Organization; WMA: World Medical Association.

#### Origin and transregional development of the therapeutic concepts

Even though empirically validations and applications did not even enter the analysis, the evaluation of the origin of the therapeutic concepts showed a complex pattern of knowledge production and a combination of multiple sources.

The studies either used already modified concepts (2, 3, 4, 8, 9, 10) and/or modified the content of the applied concept (1, 3, 6, 7, 8, 11, 12, 13, 14) and/or combined techniques from different concepts (4, 5, 6, 7). Only in two cases (SIT, U.S.; Multidisciplinary treatment based on a phase model, Germany) (2, 11) did development and modification take place in the same country, wherefore all other concepts can be considered transnationally influenced. Five studies used concepts (CA CBT, CA CBT+, KIDNET, NET) that had been previously developed or adapted for other refugee populations (2, 3, 4, 9, 10). In the case of NET, the Chilean concept of testimony therapy was in this form first modified by a German research group for treating Sudanese refugees in Uganda, and later modified as KIDNET by German and Ugandan researchers for refugee children in Uganda and by German researchers for children in Sri Lanka. Similarly, in the cases of CA CBT and CA CBT+, a U.S.-American concept was first culturally modified for Cambodian and Vietnamese refugees in the U.S. and subsequently the resulting concept was adapted by native speakers for Farsi/Dari-speaking refugees in Germany (3, 4). Additionally, five other studies used concepts (CPT, first phase of trauma-specific therapy, IITF, STARC, TF-CBT) which had been developed in the U.S. and modified in Germany (1, 6, 7, 12, 13). Of these, two concepts used techniques which are rooted in mindfulness and Buddhism (6, 7). Likewise, mindfulness techniques are used in the Imaginative Stabilization Technique, a concept that is a modification of the Germany-developed PITT (14). In the case of EMDR-based group therapy, the U.S.-American concept EMDR was adapted for groups of children by Mexican researchers in Mexico and then further modified in Germany for refugee children from different regional backgrounds. In the case of KNTT, a new concept was developed by the author for Turkish refugees in Germany. Besides containing specific knowledge about Turkish culture, the concept is influenced by U.S.-American psycholinguistic therapy and furthermore combines narrative therapy, a concept first developed in Australia and New Zealand, with the screen technique from the German concept PITT (5).

## Discussion

The review aimed at providing a theoretically as well as empirically informed basis for looking at disciplinary premises, practices, and boundaries as well as the regional locatedness of research on psychotherapy for refugees in Germany. Target groups were described, and therapeutic concepts were specifically adapted to the respective target groups in very heterogeneous ways. When target groups were restricted to participants from specific regional origins, adaptations were more likely to focus on cultural sensitivity (e.g., use of culturally relevant metaphors and examples), while studies that included refugees in general rather focused on adaptations in response to flight and migration (e.g., dealing with postmigration stress, the involvement of caregivers). Explicit transdisciplinary opening remained scarce and primarily comprised the integration of qualitative and reflexive methodology in a few cases as well as the combination of therapeutic approaches with complementary therapy. Explicit reference to anthropological and cultural theory was entirely absent. Regarding transregionality, the findings revealed a surprisingly complex pattern of development and adaptation of therapeutic concepts. Largely, however, the locus of development and adaptation appeared to be the Global North (specifically Germany and the U.S.), which is also reflected by low to very low rates of references to scholarship from the Global South.

On the more abstract level and based on the current developments around scholarship on Decolonization and GMH, the review tried to render visible how goals like “improving mental health care for refugees” or “closing the treatment gap” are inseparably tied to the concepts and methods which are used to define and approach an empirical basis. Implications that can be deduced for future empirical research involve the urgent need to reflect study aims more precisely and related to concepts and definitions of target groups and to reassess them with respect to disciplinary traditions on the one hand and knowledge from “outside the discipline” on the other. Careful attention should be paid to the sources, definition, and formation of “culture-specific knowledge.” Procedures of cultural adaptations should be described in more detail (e.g., Kananian et al., 2021), and more precise definitions of “culture-sensitivity” are needed. In view of the continuing need to decolonize science as well as psychotherapy, the range of these sources should attempt to further exceed the current focus on German- and U.S.-American scholarship. Especially studies on psychotherapy for refugees should aim at including more scholarship from the Global South as well as from countries of origin of the target groups and by scientists from these countries.

Substantially, this may result in the inclusion of indigenous disease models and healing practices, religious beliefs, and culture-specific knowledge. In the studies reviewed, it ranged from using culturally relevant metaphors and examples (as in CBT and CBT+) to adapting the narrative structure and procedure of exposure (KNTT). Regarding cultural adaptation, therapy concepts for refugees in Germany could be improved by drawing from research from countries of origin. During the last decades a considerable number of research papers have emerged which explore attitudes and perspectives towards mental health (e.g., Afghanistan: Bragin et al., 2018; Miller et al., 2008; comparison of several Arab societies: El-Islam, 2008; Rudwan & Al Owidha, 2019; Nigeria: Adewuya & Makanjuola, 2008; Gureje et al., 2005). These could provide a broad basis for informing research and practice about the role of stigma towards mental health, explanation of causes for mental disorders, and the diverging role of religious beliefs for understanding mental disorders (El-Islam, 2006). Further, and in line with this, elements of approaches to healing and therapy could be examined and possibly implemented into therapy for refugees in Germany. Depending on their background and needs, these could range from exploring the role of religious elements, like Qur’anic recitation (Ansari Jaberi et al., 2005; Babamohamadi et al., 2015), to understanding the complexity of gender roles (Bragin et al., 2018) or embedding social exchange and narration in psychotherapy (Flueckiger, 2003).

Beyond cultural adaptation, the Chilean testimony therapy is another example of how psychology can learn from approaches from the Global South. By closely connecting individual biographies to the political context, it adapts to requirements of the political situation and moves beyond the conventional boundaries of psychotherapy (Neuner et al., 2001). Nevertheless, it should be noted that not all aspects of connecting psychotherapy to the political context are unproblematic and that e.g., handing out a written document for improving the chance of getting asylum could also occasionally encourage patients to seek psychotherapy for secondary gain. Other approaches to decolonizing psychotherapy, e.g., from South Africa (Makhubela, 2016; Pillay, 2017), have shown how in certain political and historical conditions it becomes inevitable to repeatedly “ask the question of collectivity without prefabricated contents” (Spivak, 2003, p. 26). Therapists need to constantly pay attention to multiple aspects of identity as well as persisting power relations in and outside the therapy room, and on a structural level it must be asked who is selected for psychotherapy training. On the level of research, this means developing more participative research formats, which question and revise the dichotomy between “scientist” and “participant” (Wright et al., 2010). Such formats are currently rare or are conducted mostly detached from intervention studies (but see e.g., Zehetmair et al., 2019). In closer connection, they could systematically enable the integration of knowledge that is closer to the rapidly changing life situations of refugees.

Regarding the focus shift from migrants in general towards refugees as target groups, studies should be more aware of the heterogenous and complex situations and the different levels on which (flexible) adaptations need to be realized. The findings of the review imply that these needs for adaptation exceed cultural sensitivity, and for example include adaptations in response to flight and migration such as the consideration of post-migratory stress, insecure residence permits, and complex familial situations in the case of children and youths. As suggested in the studies reviewed, this could for example include crisis intervention in the case of acute postmigratory stress (Stammel et al., 2017), the development of therapy formats for children and youths which involve caregivers other than parents (Unterhitzenberger et al., 2019), and special attention to possible collective traumatization during the therapy process (Kizilhan, 2010). Furthermore, the need for intervention studies with a broader scope (e.g., disorders other than PTSD, target groups other than refugees) should be assessed. While the increase in research on psychotherapy for refugees is a positive development, it is important to further evaluate *why* other groups of migrants rarely appear in intervention studies and, if needed, to include other groups of migrants in studies.

By summarizing already existing adaptations, this review provides an informed basis for (re)combining different concepts and adaptations. Beyond the improvement of respective research, it can serve as an overview for practitioners, e.g., by informing about the various existing therapeutic concepts and the ways in which they are designed to respond to the various needs and situations of patients.

The main limitations of the review derive from the theoretical contradiction that it accepts the premises of psychology and ethnocentrism “at the very moment at which it is employed at denouncing them” (Derrida, 1999, p. 121; see also Spivak, 1994). By looking at research in this way, it already presupposed a narrow definition of science and psychology and to a certain extent cleaved to its methodologies and concepts. Besides the very methodological form and structure of a systematic review, these specifically include nation- and nationality-based terminology, the use of the concepts Global South and Global North, the blurring of possible discrepancies between epistemological and regional locatedness (Lovell et al., 2019), and the definition of interventions as psychotherapy. While research on psychotherapeutic interventions for refugees in Germany was the narrow starting point in this review, future research might as well look at other countries, or other regional entities, multinational trials, as well as other forms of mental health care, like counseling, social work, and structures of regular care. Furthermore, a particularly severe limitation to that effect is the insufficient tracing of strands of knowledge production, which significantly shape the design and outcome of an intervention study, but which are routinely rendered invisible. In detail, these include individual and personal experiences of the therapists and authors, non-written and unpublished sources of knowledge, and theoretical assumptions,which do not necessarily have to be cited or explicitly mentioned due to disciplinary practices. To tackle these issues, more collaborative and participative research is needed. Future evaluations of transdisciplinary and transregional opening could for example include dialogues with the authors, therapists, and patients, more collaborative, transregional projects, especially between researchers from the Global South and the Global North, as well as joint projects with scientists from other disciplines, like anthropology, area studies, cultural studies, or sociology.

Globalized knowledge infrastructure and increasing migration movements require psychology to genuinely reflect on its own assumptions and origins of knowledge production. At the same time, they enable new and more adaptive ways of collaboration for improving mental health care. To this end, theoretically and empirically informed reflection and recombination of already existing approaches are necessary, as well as informed and persistent striving for structural change, creativity, and openness towards the Other in clinical practice.

## Supplemental Material

sj-docx-1-tps-10.1177_13634615231187255 - Supplemental material for Research on psychotherapy for refugees in Germany: A systematic review on its transdisciplinary and transregional opening Supplemental material, sj-docx-1-tps-10.1177_13634615231187255 for Research on psychotherapy for refugees in Germany: A systematic review on its transdisciplinary and transregional opening by Annika Kleinschmitt in Transcultural Psychiatry

sj-pdf-1-tps-10.1177_13634615231187255 - Supplemental material for Research on psychotherapy for refugees in Germany: A systematic review on its transdisciplinary and transregional opening Supplemental material, sj-pdf-1-tps-10.1177_13634615231187255 for Research on psychotherapy for refugees in Germany: A systematic review on its transdisciplinary and transregional opening by Annika Kleinschmitt in Transcultural Psychiatry
