# Supplementary material for: Research on psychotherapy for refugees in Germany: A systematic review on its transdisciplinary and transregional opening
Source: Transcult Psychiatry. 2024 Jan 17;61(2):151–67. doi: 10.1177/13634615231187255 (PMC10996299; doi:10.1177/13634615231187255)
Supplement: sj-docx-1-tps-10.1177_13634615231187255 - Supplemental material for Research on psychotherapy for refugees in Germany: A systematic review on its transdisciplinary and transregional opening [file sj-docx-1-tps-10.1177_13634615231187255.docx]

**Supplementary material**

**Table 4:** The table provides an overview, by summarizing all main findings in one master table. This includes characterizations of the studies (methods, target groups and results), therapeutic concepts and their aims, origins, target group-specific adaptations and transdisciplinary elements, the total number of references of each publication and the total numbers and percentages of publications with ≥ 1 author, affiliated with an institution in the Global South. For details regarding publications, manuals, guidelines and laws published by national and international organization and institutions see Table 3.

|  | *Method* | *Target Group* | *Characterization of the target group* | *Main Result* | *Therapeutic concept and setting* | *Aims* | *Origin and target group specific adaptations of the concept^a^* | *Transdisciplinary elements* | *Number of references (total)* | *Scholarship with ≥ 1 author, affiliated with an institution in the Global South  (total/percentage)^a^* |
| --- | --- | --- | --- | --- | --- | --- | --- | --- | --- | --- |
| Brake-meier et al., 2017  [1] | Feasibility study, pilot study (*n*= 28, ITT=37) | Refugees > 18 y.,  -diagnosis of affective disorder, anxiety disorder, eating disorder somatoform disorder, or PTSD diagnosis | -traumatic experiences  -having to manage societal and vocational integration  -high prevalence of mental disorders  -legal barriers to access care system | -sign. reduction of anxiety and depression  -reduction of PTSD-symptoms n.s.  drop-out: 24 % | **IITF (Interpersonal Integrative Therapy for refugees)** *10 sessions psychotherapy, 100 min., mixed setting,*  *transdiagnostic* | Reducing symptoms and current interpersonal stress, preventing chronification, supporting integration | -U.S.-American concept (Interpersonal therapy, IPT, Klerman et al., 2004), supplemented with integration-related content by the authors  -language and culture mediation  -questionnaires in Arabic | Combined with social work (4 sessions), facultative occupational therapy (project week), psychiatric treatment | 33 | 0 |
| Hensel-Dittmann et al.,  2011 [2] | Randomized-controlled intervention study (NET: *n*=12, ITT= 15  SIT: *n*=11, ITT= 13) | Asylum seekers  -experienced organized violence  -PTSD diagnosis | -traumatic experiences  -pre, peri and post flight distress, especially continuous fear of being deported  -high prevalence of PTSD | -sign. reduction of PTSD severity only in NET group  -remission from PTSD diagnosis:  NET: 18%, SIT: 0%  drop-out:  NET: 20%, SIT: 15 % | **1. NET (Narrative exposure therapy)**  **2. SIT (Stress inoculation training)** *10 sessions, 90 min., individual setting* | NET: Emotional relief and habituation, contrasting memory and present moment SIT: Enhancing ability of coping with presently occurring stressors | NET: -based on a Chilean concept (Testimony Therapy, Lira & Weinstein) -in this version first adapted for children by (parts of) this study group for Sudanese refugees in a refugee camp in Uganda (Neuner et al., 2004)  -explores the whole biography and is therefore well suited for victims of multiple traumatic experiences  SIT: -U.S.-American concept (Meichenbaum, developed in the 1970s)  -adapted for the needs of survivors of organized violence in the U.S. (Foa, unpubl. data)  Both: -language mediation if needed | Participants received a written biography | 37 | 3/ 8.1 % Bosnia-Herzegovina (1), China (1), Uganda (1) |
| Kananian et al., 2017 [3] | Feasibility study, pilot study (*n*=7) | Farsi or Dari speaking refugees ≥18 y., male -diagnosis of trauma and stressor-related disorder, depression, anxiety disorder, or somatoform  disorder | -traumatic experiences -pre, peri and post flight distress  -unsettled life situations regarding work and residence  -local and culture specific idioms of distress  -greater emphasis on somatic symptoms | -sign. improvement of general mental health and quality of life  drop-out: 22 % | **CA CBT (Culturally Adapted Cognitive Behavioral Therapy)**  *12 sessions, 90 min., group setting, transdiagnostic* | Acceptance of and distancing from negative events, reappraisal, emotion regulation | -based on an U.S.-American concept -first developed for Cambodian (Hinton et al., 2005) and Vietnamese (Hinton et al, 2004) refugees in the U.S., modified for Farsi/Dari speaking refugees by the authors  -includes explanations of causes, metaphors, and examples from Afghan culture, additional focus on family and community  -Farsi/Dari-speaking therapists  -therapy material in Farsi/Dari, Questionnaires back-translated or validated in Farsi | Concept includes meditation and Yoga-like exercises | 44 | 8/ 18.2 % Afghanistan (1), Iran (5), Nepal (1), South Africa (1) |
| Kananian et al., 2020  [4] | Randomized-controlled pilot study (treatment group: *n*=11, ITT= 12 waiting list: *n*=12) | Farsi or Dari speaking refugees ≥18 y.,  male -diagnosis of anxiety disorder, depression, or PTSD | -traumatic experiences  -pre, peri and post flight distress  -unsettled life situations regarding work and residence  -high prevalence and comorbidity of mental disorders  -perception and expression of symptoms and conceptualizations of mental disorders differ from western culture | -sign. improvement of general mental health only in treatment group  drop-out: treatment group: 8 %  waiting list: 0 % | **CA CBT + (incl. additional problem solving training)** *12 sessions, 90 min.***,**  *group setting* | Increasing resilience to past and current sources of distress -Enhancing active coping strategies related to postmigration stressors | -based on an U.S.-American concept -first developed for Cambodian (Hinton et al., 2005) and Vietnamese (Hinton et al, 2004), modified for Farsi/Dari-speaking refugees by the authors, simplified concept -use of culturally appropriate imagery and explanations  -gender homogenous groups  -Farsi/Dari-speaking therapists -therapy material in Farsi/Dari, Questionnaires back-translated or validated in Farsi | Concept includes meditation and Yoga-like exercises | 67 | 15*^a^* / 22.4 % Afghanistan (1), China (1) India (2), Iran (4), Iraq (1), Kenya (2), Lebanon (1), Nepal (1), Pakistan (2), South Africa (1) |
| Kizilhan, 2010  [5] | Pilot study  (*n*=16) | Refugees with residence permit, from Turkey, female  -living in Germany for more than 5 years  -experienced sexual violence -PTSD diagnosis | -high prevalence of PTSD  -showing culture specific symptoms  -different health concept, no experience with psychotherapy -narration as most important element of identity in collective cultures, connects individual and collective | -sign. reduction of PTSD-related symptoms, general distress and depressive symptoms drop-out: 0% | **KNTT (Kultursensitive narrative Traumatherapie)** *12 individual sessions on average, in-patient setting* | Narrative exposure and re-integration of identity, which is fragmented due to several individual and collective traumatic experiences | -concept developed by the authors, based on narrative therapy (White & Epston, 1992^b^, Australia, New Zealand), screen technique (Reddemann, 2004, Germany) and psycholinguistic theory (Pennebaker, 2004, U.S.-America)  -different narrative structure, based on narrative position change (experiencing + emotional, observing + rational)  -includes intergenerational and collective trauma  -avoids direct exposure  -Turkish speaking therapists  -Turkish questionnaires, partly validated in Turkish | In-patient setting with individual and group therapy, physiotherapy and relaxation practice | 38 | 0 |
| Koch et al., 2020 [6] | Randomized-controlled intervention study  (treatment group: *n*=15, ITT=22 Waitlist: *n*=21, ITT=22) | Refugee youths, 15-21 y., from Afghanistan  -difficulties in emotion regulation -experienced ≥1 traumatic event | -post flight distress, like insecure residence status and structural barriers  -cultural differences  -high prevalence and comorbidity of various mental disorders | -sign. reduction of emotion-regulation difficulties and transdiagnostic symptom severity only in treatment group  drop-out: treatment group: 32%, waitlist: 5% | **STARC (Skills-Training of Affect Regulation- A culture-sensitive approach)**  *14 sessions, 90 min., group setting, transdiagnostic* | Improving emotional clarity and emotion regulation | -based on U.S.-American concepts (Skills Training in Affective and Interpersonal Regulation, STAIR, Cloitre et al., 2010; Dialectical behavioral therapy, DBT, Linehan, 2014), influenced by mindfulness/Buddhism -culturally modified by the authors  -simple language  -role clarification and non-judgmental attitude of therapist  -use of culturally relevant metaphors  -integrating relevant resource persons  -back-translated questionnaires | none | 40 | 2/ 5 % Iraq (1), Lebanon (1) |
| Kruse et al., 2009 [7] | Intervention study  (Treatment group:  *n*= 34, ITT= 35 TAU:  *n*=30, ITT= 35) | Bosnian refugees >18  -suffered severe trauma  -diagnosis of PTSD and somatoform disorder | -traumatic experiences  -pre, peri and post flight distress, especially insecure residence status -language barriers, transcultural problems  -high prevalence of (complex) PTSD and comorbidities, especially somatoform disorders | -sign. greater reduction of PTSD symptoms and general psychological distress only in treatment group  drop-out:  treatment group: 3 %, TAU: 11% | **First phase of Trauma-specific psychotherapy** *25 sessions, 50 minutes, individual setting* | Stabilization; developing feeling of safety, psychoeducation, improving affect regulation and the  ability to deal with flashbacks | U.S.-American (Courtois, 2004) /German (Reddemann, 2004) concept, influenced by mindfulness/Buddhism -adapted by the authors for this target group  -correcting culture-related convictions and worries in relation to the traumatic event  -using culturally appropriate visualization for imagination  -Bosnian speaking therapists  -one questionnaire translated into Bosnian | Increasing feeling of safety by preventing deportation during treatment | 34 | 5/ 14.7 %  Bosnia-Herzegovina (2), Lebanon (1), Mozambique (1), Uganda (1) |
| Lempertz et al., 2020 [8] | Feasibility study, pilot study  (*n*=10) | Refugee children, 4-6 years -attending a public daycare center  -parents applied for asylum -showing  posttraumatic stress response | -traumatic experiences  -pre, peri and post flight distress, especially insecure residence status -symptoms differ due to age-dependent cognitive development and language skills | -sign. decrease of PTSD score from preschool teachers´ rating, parents´ rating n.s.  Incomplete participation: 30 %, missing ratings at follow-up: parents 40 %, teachers 20% | **EMDR-Based Group therapy**  *5 sessions, 50-60 min., group setting* | Activating resources, processing flight experience and trauma, emotional habituation, developing positive future perspective | U.S.-American concept (Shapiro), Mexican adaptation (Jarero, Artigas & Montero, 2008)  -adapted for this target group by the authors -voluntary informative conversations with parents  presence of preschool teacher during sessions possible  -possibility to switch to primary language  -flight specific topics actively addressed  -example of bear as cross-cultural identification figure  -parent questionnaires translated to German, English, Arabic, Farsi, Tigrinya | Cooperation with daycare center | 36 | 1/ 2.8 %  Syria (1) |
| Neuner et al., 2010 [9] | Randomized-controlled intervention study (treatment group: *n*=14, ITT=16, TAU: *n*= 16) | Asylum-seekers and refugees  -temporary leave to remain  -history of state-sponsored  violence  -PTSD diagnosis, no comorbid disorders | -complex history of traumatic experiences -post flight stressors like unclear perspective, limited rights and access to health care  -distinct group within host countries  -cultural and language barriers  -high prevalence of PTSD | -sign. greater reduction of posttraumatic stress in treatment group  -Remission from PTSD diagnosis: 7 %  drop-out: treatment group: 13 %, TAU: 0 % | **NET (Narrative Exposure therapy)** *9 sessions, M= 120 min., individual setting* | Completion of the autobiographic memory, by activating  connections to traumatic events and representations of fear memory | -based on a Chilean concept (Testimony Therapy, Lira & Weinstein) -in this version first adapted for children by (parts of) this study group for Sudanese refugees in a refugee camp in Uganda (Neuner et al., 2004)  -explores the whole biography and is therefore well suited for victims of multiple traumatic experiences  -interpretation, female interpreters for female patients | Participants received a written biography, which they could submit to the court and/or human rights organization | 40 | 5/ 12.5 % Bosnia-Herzegovina (1), Chile (1), China (1), Malaysia (1), Sri Lanka (1) |
| Ruf et al., 2010 [10] | Randomized-controlled intervention study (treatment group:  *n*= 12, ITT=13 waitlist: *n*=13) | Refugee children, 7-16 years -PTSD diagnosis  -possibly comorbid disorders | -multiple traumatic experiences -high risk of mental disorders, especially PTSD and associated functional impairment (e.g., problems in school, barriers to integration) | -sign. reduction of PTSD symptoms and severity only in treatment group -sustainable effects after 12 months drop-out: treatment group: 8 %  waiting list: 0 % | **KIDNET (narrative exposure therapy for children)**  *10 sessions, 90-120 min., individual setting* | Constructing a chronological narrative of the whole life, incl. traumatic events, countering avoidance and recovering the full implicit information of the traumatic experience | -based on a Chilean concept (Testimony Therapy, Lira & Weinstein) - In this version first adapted for children by (parts of) this study group for refugee children in Uganda (Onyut et al., 2005; Schauer et al., 2004) and children in Sri Lanka (Catani et al., 2009)  -facultative translation -involvement of parents not necessary | Child receives document, which may be used for children rights advocacy or asylum process | 43 | 6/ 14 %  Chile (1), Iran (1), Sri Lanka (1), Uganda (3) |
| Stammel et al., 2017 [11] | Treatment study in naturalistic setting, single group setting (*n*= 76, ITT= 167) | Refugees and asylum seekers  -suffering from torture or war-related violence -severe and complex symptomatology | -traumatic experiences -postmigration stress (e.g., asylum regulation  problems, uncertainty, difficult  living and social conditions)  -high prevalence of mental disorders, especially PTSD  -limited access to health care | -sign. reduction of trauma-related distress, somatoform symptoms and depression -increase of quality of life  drop-out: 55 % | **Multidiscipli-nary treatment, based on a phase model**  *One individual therapy session per week, combination of group and individual setting, in-patient setting* | Stabilization, exposure-based processing of traumatic experience and dealing with trauma-related symptoms, developing future perspective, relapse prevention | -German concept, phase model (Wenk-Ansohn et al., 2014; Gurris & Wenk-Ansohn, 2013)  -adaptation by the treatment providers in response to individual needs  -assisted by interpreters  -crisis intervention in case of acute post-migratory stress  -(back)translated questionnaires | Culturally sensitive medical, psychiatric and social treatment services, supplemented by body and creative therapeutic modules | 54 ^b^ | 1/ 1.9 % Nepal (1) |
| Steil et al, 2021 [12] | Feasibility study,  Pilot study,  Mixed methods  (*n*=7, ITT=16) | Refugees ≥ 18 y.,  -unsafe country of origin  -min. temporary residence permit  -PTSD diagnosis | -traumatic experiences  -post flight distress (e.g., insecure residence status, unstable living conditions)  -low literacy rate | -reduction of PTSD symptoms n.s.  drop-out: 56 % | **CPT (Cognitive processing therapy)**  *17-21 sessions, 100 min., individual setting* | Psychoeducation, cognitive restructuring, belief modification, stabilization | -U.S.-American concept (Resick et al., 2017), slightly adapted by the authors  -therapists received training in language-mediated therapy and in dealing with culture-specific particularities  -therapy material in Arabic and Persian  -includes examples, which are typical for refugees  -facultative language mediation | Cooperation with volunteer workers | 50 | 4/ 8 % China (1), Congo (1), Iraq (2) |
| Unterhitzenberger et al., 2019 [13] | Feasibility study, pilot study (*n*= 19, ITT=22) | URM (Unaccompanied refugee minors), <21 years -PTSD diagnosis -living in youth welfare facility -availability of a caregiver | -traumatic experiences  -pre, peri and post flight distress, especially uncertain residence status, lack of social support  -high prevalence of mental disorders  -different concepts of mental health and treatment -barriers to access health care system | -sign. decrease of PTSD symptoms  - 84% of PTSD cases recovered after treatment  drop-out: 15 % | **TF-CBT (Trauma focused cognitive behavioral therapy)** *On average 15 sessions, 100 min., 8 of which with caregiver,*  *Individual setting* | Acquiring stabilization skills, narrative, and cognitive processing of trauma, enhancing safety, integrating trauma in one´s life | U.S.- American concept (Cohen et al., 2017), adapted by the authors  -high level of caregiver involvement  -facultative language and culture mediation -possibility to choose interpreter’s gender  -facultative grief specific components  -developing safety plan for possible refusal of asylum -adding URM-specific items to trauma questionnaire | Involvement of caregivers | 39 | 1/ 2.6 % Zambia (1) |
| Zehetmair et al., 2018 [14] | Feasibility study, pilot study (*n*= 17, ITT= 46) | Refugee youth < 18 y., male, English-speaking  -currently living in reception center, applied for asylum or currently in the process of  -PTSD diagnosis | -pre, peri and post flight distress  -no stable living conditions  -high risk for developing mental illness | -increased perception of positive feelings and being in control, reduced distress and anxiety symptoms  -reduction of arousal, depressive and PTSD symptoms n.s. drop-put/incomplete participation: 57 % | **Imaginative stabilization techniques** *Open group setting, min. 4 sessions attended, M = 5,43, range 4-40* | Developing, activating, strengthening individual skills, resources + coping strategies | -based on German concept (Psychodynamic Imaginative Trauma Therapy, PITT, Reddemann, 2017), influenced by mindfulness/Buddhism  -adapted by the authors -cross-culturally adapted questionnaires  -therapy in English | none | 71^b^ | 7/ 9.9% Bosnia-Herzegovina (1), El Salvador (1), Iran (1), Iraq (2), South Africa (1), Uganda (1) |
