# Supplementary material for: Research on psychotherapy for refugees in Germany: A systematic review on its transdisciplinary and transregional opening
Source: Transcult Psychiatry. 2024 Jan 17;61(2):151–67. doi: 10.1177/13634615231187255 (PMC10996299; doi:10.1177/13634615231187255)
Supplement: sj-pdf-1-tps-10.1177_13634615231187255 - Supplemental material for Research on psychotherapy for refugees in Germany: A systematic review on its transdisciplinary and transregional opening [file sj-pdf-1-tps-10.1177_13634615231187255.pdf]

Supplementary material

Table 1: Characterizations of the studies: methods, target groups and results

Study number	*Study*	*Method*	*Target Group*	*Characterization of the target group*	*Main Result*
**1**	Brakemeier et al., 2017	Feasibility study, pilot study (*n*= 28, ITT=37)	Refugees > 18 y., -diagnosis of affective disorder, anxiety disorder, eating disorder somatoform disorder, or PTSD diagnosis	-traumatic experiences<br>-having to manage societal and vocational integration<br>-high prevalence of mental disorders<br>-legal barriers to access care system	-sign. reduction of anxiety and depression<br>-reduction of PTSD-symptoms n.s.<br>-drop-out: 24 %
**2**	Hensel-Dittmann et al., 2011	Randomized-controlled intervention study (NET: *n*=12, ITT= 15 SIT: *n*=11, ITT= 13)	Asylum seekers<br>-experienced organized violence<br>-PTSD diagnosis	-traumatic experiences<br>-pre, peri and post flight distress, especially continuous fear of being deported<br>-high prevalence of PTSD	-sign. reduction of PTSD severity only in NET group<br>-remission from PTSD diagnosis: NET: 18%, SIT: 0%<br>drop-out:<br>NET: 20%, SIT: 15 %
**3**	Kananian et al., 2017	Feasibility study, pilot study (*n*=7)	Farsi or Dari speaking refugees ≥18 y., male<br>-diagnosis of trauma and stressor-related disorder, depression, anxiety disorder, or somatoform disorder	-traumatic experiences<br>-pre, peri and post flight distress<br>-unsettled life situations regarding work and residence<br>-local and culture specific idioms of distress<br>-greater emphasis on somatic symptoms	-sign. improvement of general mental health and quality of life<br>-drop-out: 22 %
**4**	Kananian et al., 2020	Randomized-controlled pilot study (treatment group: *n*=11, ITT= 12 waiting list: *n*=12)	Farsi or Dari speaking refugees ≥18 y., male<br>-diagnosis of anxiety disorder, depression, or PTSD	-traumatic experiences<br>-pre, peri and post flight distress<br>-unsettled life situations regarding work and residence<br>-high prevalence and comorbidity of mental disorders<br>-perception and expression of symptoms and conceptualizations of mental disorders differ from western culture	-sign. improvement of general mental health only in treatment group<br>-drop-out:<br>treatment group: 8 %<br>waiting list: 0 %
**5**	Kizilhan, 2010	Pilot study (*n*=16)	Refugees with residence permit, from Turkey, female<br>-living in Germany for more than 5 years<br>-experienced sexual violence<br>-PTSD diagnosis	-high prevalence of PTSD<br>-showing culture specific symptoms<br>-different health concept, no experience with psychotherapy<br>-narration as most important element of identity in collective cultures, connects individual and collective	-sign. reduction of PTSD-related symptoms, general distress and depressive symptoms<br>-drop-out: 0%
**6**	Koch et al., 2020	Randomized-controlled intervention study (treatment	Refugee youths, 15-21 y., from Afghanistan<br>-difficulties in emotion regulation	-post flight distress, like insecure residence status and structural barriers<br>-cultural differences	-sign. reduction of emotion-regulation difficulties and transdiagnostic symptom severity

Study number	Study	Method	Target Group	Characterization of the target group	Main Result
		group: *n*=15, ITT=22 Waitlist: *n*=21, ITT=22)	-experienced ≥1 traumatic event	-high prevalence and comorbidity of various mental disorders	only in treatment group<br>-drop-out: treatment group: 32%, waitlist: 5%
**7**	Kruse et al., 2009	Intervention study (Treatment group: *n*= 34, ITT= 35 TAU: *n*=30, ITT= 35)	Bosnian refugees >18<br>-suffered severe trauma<br>-diagnosis of PTSD and somatoform disorder	-traumatic experiences<br>-pre, peri and post flight distress, especially insecure residence status<br>-language barriers, transcultural problems<br>-high prevalence of (complex) PTSD and comorbidities, especially somatoform disorders	-sign. greater reduction of PTSD symptoms and general psychological distress only in treatment group<br>-drop-out: treatment group: 3 %, TAU: 11%
**8**	Lempertz et al., 2020	Feasibility study, pilot study (*n*=10)	Refugee children, 4-6 years<br>-attending a public daycare center<br>-parents applied for asylum<br>-showing posttraumatic stress response	-traumatic experiences<br>-pre, peri and post flight distress, especially insecure residence status<br>-symptoms differ due to age-dependent cognitive development and language skills	-sign. decrease of PTSD score from preschool teachers´ rating, parents´ rating n.s. Incomplete participation: 30 %, missing ratings at follow-up: parents 40 %, teachers 20%
**9**	Neuner et al., 2010	Randomized -controlled intervention study (treatment group: *n*=14, ITT=16, TAU: *n*= 16)	Asylum-seekers and refugees<br>-temporary leave to remain<br>-history of state-sponsored violence<br>-PTSD diagnosis, no comorbid disorders	-complex history of traumatic experiences<br>-post flight stressors like unclear perspective, limited rights and access to health care<br>-distinct group within host countries<br>-cultural and language barriers<br>-high prevalence of PTSD	-sign. greater reduction of posttraumatic stress in treatment group<br>-Remission from PTSD diagnosis: 7 %<br>-drop-out: treatment group: 13 %, TAU: 0 %
**10**	Ruf et al., 2010	Randomized -controlled intervention study (treatment group: *n*= 12, ITT=13 waitlist: *n*=13)	Refugee children, 7-16 years<br>-PTSD diagnosis<br>-possibly comorbid disorders	-multiple traumatic experiences<br>-high risk of mental disorders, especially PTSD and associated functional impairment (e.g., problems in school, barriers to integration)	-sign. reduction of PTSD symptoms and severity only in treatment group<br>-sustainable effects after 12 months<br>-drop-out: treatment group: 8 %<br>waiting list: 0 %
**11**	Stammel et al., 2017	Treatment study in naturalistic setting, single group setting (*n*= 76, ITT= 167)	Refugees and asylum seekers<br>-suffering from torture or war-related violence<br>-severe and complex symptomatology	-traumatic experiences<br>-postmigration stress (e.g., asylum regulation problems, uncertainty, difficult living and social conditions)<br>-high prevalence of mental disorders, especially PTSD<br>-limited access to health care	-sign. reduction of trauma-related distress, somatoform symptoms and depression<br>-increase of quality of life<br>-drop-out: 55 %

Study number	Study	Method	Target Group	Characterization of the target group	Main Result
**12**	Steil et al, 2021	Feasibility study, Pilot study, Mixed methods (*n*=7, ITT=16)	Refugees ≥ 18 y.,<br>-unsafe country of origin<br>-min. temporary residence permit<br>-PTSD diagnosis	-traumatic experiences<br>-post flight distress (e.g., insecure residence status, unstable living conditions)<br>-low literacy rate	-reduction of PTSD symptoms n.s.<br>-drop-out: 56 %
**13**	Unterhitzenberger et al., 2019	Feasibility study, pilot study (*n*= 19, ITT=22)	URM (Unaccompanied refugee minors), <21 years<br>-PTSD diagnosis<br>-living in youth welfare facility<br>-availability of a caregiver	-traumatic experiences<br>-pre, peri and post flight distress, especially uncertain residence status, lack of social support<br>-high prevalence of mental disorders<br>-different concepts of mental health and treatment<br>-barriers to access health care system	-sign. decrease of PTSD symptoms<br>- 84% of PTSD cases recovered after treatment<br>-drop-out: 15 %
**14**	Zehetmair et al., 2018	Feasibility study, pilot study (*n*= 17, ITT= 46)	Refugee youth < 18 y., male, English-speaking<br>-currently living in reception center, applied for asylum or currently in the process of<br>-PTSD diagnosis	-pre, peri and post flight distress<br>-no stable living conditions<br>-high risk for developing mental illness	-increased perception of positive feelings and being in control, reduced distress and anxiety symptoms<br>-reduction of arousal, depressive and PTSD symptoms n.s.<br>-drop-out/ incomplete participation: 57 %

Abbreviations: ITT, Intent-to-treat; *n*, number of participants*;* n.s., not significant; NET, Narrative Exposure Therapy; PTSD, Posttraumatic Stress Disorder; SIT, Stress Inoculation Training, TAU, Treatment-as-usual

Table 2: Therapeutic concepts and their aims, origins, target group-specific adaptations and transdisciplinary elements

Study number	Study	Therapeutic concept and setting	Aims	Origin and target group specific adaptations of the concept[a]	Transdisciplinary elements
**1**	Brake-meier et al., 2017	**IITF (Interpersonal Integrative Therapy for refugees)**<br>*10 sessions psychotherapy*	Reducing symptoms and current interpersonal stress, preventing chronification,	-U.S.-American concept (Interpersonal therapy, IPT, Klerman et al., 1984), supplemented with integration-related content by the authors<br>-language and culture mediation<br>-questionnaires in Arabic	Combined with social work (4 sessions), facultative occupational

Study number	*Study*	*Therapeutic concept and setting*	*Aims*	*Origin and target group specific adaptations of the concept*[a]	*Transdisciplinary elements*
		, 100 min., mixed setting, Transdiagnostic	supporting integration		therapy (project week), psychiatric treatment
**2**	Hensel-Dittmann et al., 2011	**1. NET (Narrative exposure therapy)**<br>**2. SIT (Stress inoculation training)**<br>*10 sessions, 90 min., individual setting*	NET: Emotional relief and habituation, contrasting memory and present moment<br>SIT: Enhancing ability of coping with presently occurring stressors	NET: -based on a Chilean concept (Testimony Therapy, Lira & Weinstein)<br>-in this version first adapted for children by (parts of) this study group for Sudanese refugees in a refugee camp in Uganda (Neuner et al., 2004)<br>-explores the whole biography and is therefore well suited for victims of multiple traumatic experiences<br>SIT: -U.S.-American concept (Meichenbaum, developed in the 1970s)<br>-adapted for the needs of survivors of organized violence in the U.S. (Foa, unpubl. data)<br>Both: -language mediation if needed	Participants received a written biography
**3**	Kananian et al., 2017	**CA CBT (Culturally Adapted Cognitive Behavioral Therapy)**<br>*12 sessions, 90 min., group setting, transdiagnostic*	Acceptance of and distancing from negative events, reappraisal, emotion regulation	-based on an U.S.-American concept<br>-first developed for Cambodian (Hinton et al., 2005) and Vietnamese (Hinton et al, 2004) refugees in the U.S., modified for Farsi/Dari speaking refugees by the authors<br>-includes explanations of causes, metaphors, and examples from Afghan culture, additional focus on family and community<br>-Farsi/Dari-speaking therapists<br>-therapy material in Farsi/Dari, Questionnaires back-translated or validated in Farsi	Concept includes meditation and Yoga-like exercises
**4**	Kananian et al., 2020	**CA CBT + (incl. additional problem solving training)**<br>*12 sessions, 90 min.***,** *group setting*	Increasing resilience to past and current sources of distress<br>-Enhancing active coping strategies related to postmigration stressors	-based on an U.S.-American concept<br>-first developed for Cambodian (Hinton et al., 2005) and Vietnamese (Hinton et al, 2004), modified for Farsi/Dari-speaking refugees by the authors, simplified concept<br>-use of culturally appropriate imagery and explanations<br>-gender homogenous groups<br>-Farsi/Dari-speaking therapists<br>-therapy material in Farsi/Dari, Questionnaires back-translated or validated in Farsi	Concept includes meditation and Yoga-like exercises
**5**	Kizilhan, 2010	**KNTT (Kultursensitive narrative Traumatherapie)**<br>*12 individual sessions on average, in-patient setting*	Narrative exposure and re-integration of identity, which is fragmented due to several individual and collective traumatic experiences	-concept developed by the authors, based on narrative therapy (White & Epston, 1992[b], Australia, New Zealand), screen technique (Reddemann, 2004, Germany) and psycholinguistic theory (Pennebaker, 2004, U.S.-America)<br>-different narrative structure, based on narrative position change (experiencing + emotional, observing + rational)<br>-includes intergenerational and collective trauma<br>-avoids direct exposure	In-patient setting with individual and group therapy, physiotherapy and relaxation practice

Study number	*Study*	*Therapeutic concept and setting*	*Aims*	*Origin and target group specific adaptations of the concept[a]*	*Transdisciplinary elements*
				-Turkish speaking therapists<br>-Turkish questionnaires, partly validated in Turkish	
**6**	Koch et al., 2020	**STARC (Skills-Training of Affect Regulation- A culture-sensitive approach)**<br>*14 sessions, 90 min., group setting, transdiagnostic*	Improving emotional clarity and emotion regulation	-based on U.S.-American concepts (Skills Training in Affective and Interpersonal Regulation, STAIR, Cloitre et al., 2010; Dialectical behavioral therapy, DBT, Linehan, 2014), influenced by mindfulness/Buddhism<br>-culturally modified by the authors<br>-simple language<br>- a non-judgmental attitude of therapist, encouraging to take the cultural contexts into account before judging a behavior<br>-use of culturally relevant metaphors<br>-integrating relevant resource persons<br>-back-translated questionnaires	none
**7**	Kruse et al., 2009	**First phase of Trauma-specific psychotherapy**<br>*25 sessions, 50 minutes, individual setting*	Stabilization; developing feeling of safety, psychoeducation, improving affect regulation and the ability to deal with flashbacks	U.S.-American (Courtois, 2004) /German (Reddemann & Dehner-Rau, 2004) concept, influenced by mindfulness/Buddhism<br>-adapted by the authors for this target group<br>-correcting culture-related convictions and worries in relation to the traumatic event<br>-using culturally appropriate visualization for imagination<br>-Bosnian speaking therapists<br>-one questionnaire translated into Bosnian	Increasing feeling of safety by preventing deportation during treatment
**8**	Lempertz et al., 2020	**EMDR-Based Group therapy**<br>*5 sessions, 50-60 min., group setting*	Activating resources, processing flight experience and trauma, emotional habituation, developing positive future perspective	U.S.-American concept (Shapiro), Mexican adaptation (Jarero, Artigas & Montero, 2008)<br>-adapted for this target group by the authors<br>-voluntary informative conversations with parents<br>presence of preschool teacher during sessions possible<br>-possibility to switch to primary language<br>-flight specific topics actively addressed<br>-example of bear as cross-cultural identification figure<br>-parent questionnaires translated to German, English, Arabic, Farsi, Tigrinya	Cooperation with daycare center
**9**	Neuner et al., 2010	**NET (Narrative Exposure therapy)**<br>*9 sessions, M= 120 min., individual setting*	Completion of the autobiographic memory, by activating connections to traumatic events and representations of fear memory	-based on a Chilean concept (Testimony Therapy, Lira & Weinstein)<br>-in this version first adapted for children by (parts of) this study group for Sudanese refugees in a refugee camp in Uganda (Neuner et al., 2004)<br>-explores the whole biography and is therefore well suited for victims of multiple traumatic experiences<br>-interpretation, female interpreters for female patients	Participants received a written biography, which they could submit to the court and/or human rights organization

Study number	*Study*	*Therapeutic concept and setting*	*Aims*	*Origin and target group specific adaptations of the concept*[a]	*Transdisciplinary elements*
**10**	Ruf et al., 2010	**KIDNET (narrative exposure therapy for children)** *10 sessions, 90-120 min., individual setting*	Constructing a chronological narrative of the whole life, incl. traumatic events, countering avoidance and recovering the full implicit information of the traumatic experience	-based on a Chilean concept (Testimony Therapy, Lira & Weinstein)<br>- In this version first adapted for children by (parts of) this study group for refugee children in Uganda (Onyut et al., 2005) and children in Sri Lanka (Catani et al., 2009)<br>-facultative translation<br>-involvement of parents not necessary	Child receives document, which may be used for children rights advocacy or asylum process
**11**	Stammel et al., 2017	**Multidiscipli-nary treatment, based on a phase model** *One individual therapy session per week, combination of group and individual setting, in-patient setting*	Stabilization, exposure-based processing of traumatic experience and dealing with trauma-related symptoms, developing future perspective, relapse prevention	-German concept, phase model (Gurris & Wenk-Ansohn, 2013)<br>-adaptation by the treatment providers in response to individual needs<br>-assisted by interpreters<br>-crisis intervention in case of acute post-migratory stress<br>-(back)translated questionnaires	Culturally sensitive medical, psychiatric and social treatment services, supplemented by body and creative therapeutic modules
**12**	Steil et al, 2021	**CPT (Cognitive processing therapy)** *17-21 sessions, 100 min., individual setting*	Psychoeducation, cognitive restructuring, belief modification, stabilization	-U.S.-American concept (Resick et al., 2017), slightly adapted by the authors<br>-therapists received training in language-mediated therapy and in dealing with culture-specific particularities<br>-therapy material in Arabic and Persian<br>-includes examples, which are typical for refugees<br>-facultative language mediation	Cooperation with volunteer workers
**13**	Unterhitzenberger et al., 2019	**TF-CBT (Trauma focused cognitive behavioral therapy)** *On average 15 sessions, 100 min., 8 of which with caregiver, Individual setting*	Acquiring stabilization skills, narrative, and cognitive processing of trauma, enhancing safety, integrating trauma in one´s life	U.S.- American concept (Cohen et al., 2017), adapted by the authors<br>-high level of caregiver involvement<br>-facultative language and culture mediation<br>-possibility to choose interpreter's gender<br>-facultative grief specific components<br>-developing safety plan for possible refusal of asylum<br>-adding URM-specific items to trauma questionnaire	Involvement of caregivers
**14**	Zehetmair et al., 2018	**Imaginative stabilization techniques** *Open group setting, min. 4 sessions attended, M =*	Developing, activating, strengthening individual skills, resources + coping strategies	-based on German concept (Psychodynamic Imaginative Trauma Therapy, PITT, Reddemann, 2017), influenced by mindfulness/Buddhism<br>-adapted by the authors<br>-cross-culturally adapted questionnaires<br>-therapy in English	none

Study number	*Study*	*Therapeutic concept and setting*	*Aims*	*Origin and target group specific adaptations of the concept*[a]	*Transdisciplinary elements*
		5,43, range 4-40			

[a] Unless otherwise indicated, sources are directly retracted from the respective study and do not appear in the list of references of this study

[b] Source is added based on literature search

Abbreviations: M, arithmetic mean; U.S., United States of America
